# Assessing the Validity of Front-of-Pack Nutrition Labels for Evaluating the Healthiness of Mediterranean Food Choices: A Global Comparison

**DOI:** 10.3390/nu16172925

**Published:** 2024-09-01

**Authors:** Julia Fernandez-Alonso, María del Mar Lamas-Mendoza, Nidia Rodriguez-Sanchez, Stuart D. R. Galloway, Leyre Gravina

**Affiliations:** 1 Nursing I Department, Faculty of Medicine and Nursing, University of the Basque Country UPV/EHU, Bº Sarriena s/n, 48940 Leioa, Spain; julia.fernandez@ehu.eus (J.F.-A.); mariadelmar.lamasmendoza@osakidetza.eus (M.d.M.L.-M.); 2 Biobizkaia Basque Health Research Institute, 48903 Barakaldo, Spain; 3 Osakidetza Basque Health Service, Bilbao-Basurto Integrated Healthcare Organisation, Basurto University Hospital, 48013 Bilbao, Spain; 4 Physiology, Exercise and Nutrition Research Group, Faculty of Health Sciences and Sport, University of Stirling, Stirling FK9 4LA, UK; nidia.rodriguezsanchez@stir.ac.uk (N.R.-S.); s.d.r.galloway@stir.ac.uk (S.D.R.G.)

**Keywords:** front-of-pack label, Nutri-score, food labelling, healthy food choice, warning labels, traffic light labels

## Abstract

In response to growing public health concerns, governments worldwide have implemented various nutrition labelling schemes to promote healthier eating habits. This study aimed to assess the consistency and effectiveness of these labels in an out-of-home context, specifically focusing on restaurant, hospitality, and institutional food service settings. In total, 178 different dishes from Spain were analysed using labels from the Mazocco method, the UK’s traffic light system, the Health Star Rating (Australia), Nutri-Score (France), multiple traffic lights (Ecuador), and warning labels (Chile and Uruguay). The results demonstrated a generally low level of agreement among these labels (K < 0.40), indicating notable variability and a lack of consensus, which could hinder consumers’ ability to make informed food choices in out-of-home settings. Nutri-Score classified the highest number of dishes as unhealthy (38%). This study underscores the need for an easy-to-understand labelling system tailored to each country’s culinary and socio-cultural contexts to improve consumer decision-making in various dining environments. Future research should focus on developing and testing qualitative methods to more accurately gauge the nutritional quality of cooked dishes in diverse out-of-home settings, thereby enhancing public health outcomes. By addressing the specific needs of the home, restaurants, hospitality, and institutional food services, tailored labelling schemes could significantly improve consumers’ ability to make healthier food choices.

## 1. Introduction

An unhealthy diet, characterised by high levels of sodium, sugar, and saturated fatty acids (SFAs), is a well-established risk factor for obesity and numerous other health problems [1,2]. In recent years, the prevalence of overweight and obesity has increased, and this has been linked to an increase in meals eaten away from home [3]. Various nutrition labels have been developed worldwide to promote healthier food choices when eating away from home, aimed at helping consumers reduce their intake of these unhealthy components. Front-of-Pack (FOP) labelling, in particular, has been shown to be an effective tool in enhancing consumer understanding and facilitating healthier food choices [4], demonstrating that FOP labels can potentially improve public health in the long term [5,6,7].

In that sense, the FOP labelling of foods provides consumers easy access to essential nutritional information, regardless of their lack of previous nutrition knowledge. However, there is a great controversy regarding the optimal design of FOP labels [8]. Each FOP label can use a different nutrient profiling system, designed to assess the nutritional value of foods based on various criteria and algorithms that consider different nutritional components and ingredients. These systems can vary in how they weigh and categorize the nutrients, leading to differences in how the FOP labelling presents the nutritional information on food products. These variations allow the labels to be tailored to each labelling system’s specific contexts and objectives. Various studies suggest that the FOP labels that use colour coding, such as the traffic light system used in the United Kingdom (UK), are more easily understood by consumers [8,9,10,11,12]. This FOP label system assigns green, amber, and red colours to a dish or food, with green being the healthiest, based on its fat, SFAs, sugars, or salt [13]. Other authors claim that the Latin American warning labels, which use a black seal to indicate the presence of excessive salt and SFAs, among other components, are equally effective and easy to read [8,10,14]. Research indicates that these labels are highly effective tools for identifying unhealthy food products due to the high presence of substances such as fat and sugars. Moreover, the absence of this indicator is sufficient and helpful for consumers to identify healthier options [15] and to be effective in encouraging food choices [16]. In Europe, the Nutri-Score system has been established in recent years, and it uses both letters and colours to evaluate food and drink [6,17]. This FOP label has been the subject of many published scientific studies demonstrating its efficacy, relevance, and usefulness for consumers and public health [18]. In Oceania, the Health Star Rating (HSR) system assigns a score using stars, where a higher number of stars indicates greater healthiness [19,20]. This system encourages the reformulation of processed products, making them healthier, particularly reductions in sodium and increases in dietary fibre [21].

These FOP labels are based on Codex Alimentarius guidelines to ensure that they are relevant and effective in specific cultural and regional contexts [22]. The Codex Alimentarius follows standards that ensure foods are safe and can be delivered to consumers. However, these FOP systems have mainly been used for pre-packaged food sold in shops or processed products [23] and not in the context of meals away from home. The extension of these principles to cooked dishes catered at any food service, where the consumer can choose which one to consume from among several offered, needs to be thoroughly investigated as it is emerging as an area of interest. Thus, a recent study by Yang Y et al. (2024) evaluated the FOP labels in online meal services and explored their potential application in the food service sector [24]. Another well-known intervention in away-from-home dining establishments is menu labelling, which consists of making nutritional information about the foods served available at the point of ordering or purchase [25]. This has been associated with serving lower fat and lower salt items in popular UK chain restaurants [26]. Moreover, menu labelling policies have been implemented in chain restaurants, usually focusing on displaying the energy content [27]. However, calorie labelling on the foods purchased or consumed in real-world settings showed mixed results in several systematic reviews, concluding that their impacts are limited [28,29,30]. Nevertheless, although Food-Based Dietary Guidelines exist to inform consumer behaviour regarding meals and cooked dishes, the planning, political, and financial support for their implementation in different sectors, such as restaurants and food services, are still insufficient, limiting their real impact [31]. Additionally, an international comparative analysis of the dietary guidelines from various countries shows that, while all provide recommendations on fruits, vegetables, cereals, legumes, dairy, meats, and oils, there are notable differences in the recommended amounts. These variations underscore the need to adapt the guidelines to specific cultural and regional contexts to make them practical and relevant [32]. There is still no national policy on health logos on menus worldwide, but some have started to put sodium warning icons next to restaurant menu items that contain high sodium values [33]. Knowing that assessing the nutritional value of cooked dishes often requires complex, quantitative, and time-consuming methods, such as weighing ingredients and analysing their nutritional content through specific nutritional software [34,35], there is still the need for a clear and easily understandable system for labelling the healthfulness of cooked dishes to guide consumers’ food choices and contribute to health outcomes [36]. Therefore, considering the significant impact these interventions can have on consumer choice and health, FOP labelling could serve as a valuable tool for providing more detailed information about the nutritional quality of the dishes chosen by consumers. Implementing FOP labels on prepared and cooked meals, especially in out-of-home settings such as restaurants, hospitality, and institutional food services, can offer consumers an accessible tool to assess the healthiness of their meal choices. This approach not only facilitates more informed decision-making but also has the potential to promote healthier eating habits and positively impact public health outcomes.

Therefore, to determine the relationship between the different weightings and categorizations of the nutrients for the same observed food or dish in out-of-home contexts, we propose using nutrition labelling resources, such as the FOP labels implemented in several countries. Such analysis could provide more reliable information about the healthiness of different dishes and facilitate the comparison and adaptation of international labelling systems to local contexts, ensuring accurate assessments of the food offered and consumed in settings like restaurants and institutional food services. Although there has been some research on the effectiveness of these labelling systems based on consumer opinions [9,10,11,19] and food professionals [6], studies comparing the concordance of FOP labels applied to different cooked dishes are scarce. Thus, this study aims to compare the degree of agreement of various FOP labels used globally to identify the most reliable FOP labels for evaluating the nutritional quality of dishes in out-of-home contexts and helping consumers make informed decisions about the healthiness of their chosen meals.

## 2. Methods

### 2.1. Design and Sample

In this descriptive cross-sectional study, many dishes offered by the School of Food and Catering at a university campus in Spain were chosen to analyse their nutritional quality. The dishes offered during a randomly selected week each month across ten months were considered. In total, 178 different dishes were analysed, divided into 68 main courses, 59 second courses, and 51 desserts. The nutritional quality of each course was analysed and compared using several methods: the method based on the study by Mazocco et al. [12]; the UK’s traffic light labelling system (from the UK) [37]; the Health Star Rating (HSR; Australia) [20]; Nutri-Score (France) [7]; multiple traffic lights (Ecuador) [38]; and warning seals, including those from Chile [2] and Uruguay [39]. Table 1 presents the different FOP labels analysed (all valid for cooked dishes), the variables they study, and their respective visualisation methods. This study did not require approval from any ethics committee as it used published secondary data, and no data involved human data.

### 2.2. Calculation of the Weight of the Dishes

To calculate the FOP labels, it was necessary to estimate the weight of each dish. The website https://jatondo.hostelerialeioa.net/es/canteens/txopitea (accessed on 1 June 2020) was used, indicating each dish’s menus, recipes, and ingredients. The ingredients per serving were added up, and the weight variations of the food during cooking were considered and calculated [40].

### 2.3. Dish Evaluation Tools

#### 2.3.1. Mazocco Method

This labelling system classifies the whole plate following the same guidelines as the UK traffic light system [12]. Specifically, this evaluation classifies the energy density and Na content into high energy density (from 4 to 9 kcal/g), medium energy density (from 1.5 to 4 kcal/g), low energy density (from 0.7 to 1.5 kcal/g), and very low energy density (from 0 to 0.6 kcal/g). Subsequently, according to the criteria of the “UK Food Standards Agency” [13], it classifies the Na concentration per 100 g into high Na content (>600 mg Na), medium Na content (from 120 to 600 mg Na), and low Na content (≤120 mg Na). Following both criteria, each level (high, medium, and low) is assigned, like UK traffic lights, with a colour (red, amber, and green, respectively). The final colour of the dish follows the following pattern: if the dish has two categories with the same colour, that colour is established as the predominant colour of the dish, but, in the case of two colours (green and yellow, green and red, or yellow and red), the colour that is furthest from indicating that the dish is healthy is considered to predominate. The colours were subsequently replaced by 1, 2.5, or 5 depending on whether they were red, yellow or green, respectively, and the mean was calculated.

#### 2.3.2. UK’s Traffic Light Labelling System

This guide (from now on “UK”) was developed to support communication and information to the consumer through nutritional labelling with colours: green, amber, or red, green being the healthiest and red the least healthy [41]. The parameters evaluated are the energy in Kcal and Kj per 100 g or ml per serving. As the dishes are cooked, fat receives a green score if it contains ≤ 3.0 g/100 g, yellow if it contains > 3 g to ≤17.5 g/100 g, and red > 17.5 g/100 g. The SFAs are green at ≤1.5 g/100 g, yellow from >1.5 g to ≤5 g/100 g, and red if >25%. The sugars receive a green with ≤5.0 g/100 g, yellow with >5 g to ≤22.5 g/100 g, or red with >22.5 g/100 g. Finally, salt has a green if ≤0.3 g/100 g, yellow with >0.3 g to ≤1.5 g/100 g, and red if >1.5 g/100 g [37]. Subsequently, the colours were replaced by 1, 2.5, or 5 depending on whether they were red, yellow, or green, respectively, and the arithmetic mean was taken.

#### 2.3.3. Health Star Rating (HSR)

The HSR is a labelling system that has been implemented by the Australian government in conjunction with industry, public health, and various consumer organisations since 2014. The algorithm rates the nutritional profile of packaged foods and assigns them a rating from ½ star to 5 stars (points) in 10 increments of half a star each. Thus, both “risk” components (total energy, total sugars, SFAs, and Na) and “healthy” components (fibre, fruit and vegetable content, nuts, legumes, and protein) are evaluated. The more stars the food has, the healthier the consumer’s food choice [20]. In this case, being a single numeric variable, it was unnecessary to substitute the result or calculate the mean.

#### 2.3.4. Nutri-Score

Nutri-Score labelling is represented by a five-colour scale (dark green, light green, yellow, light orange, and red or dark orange) [11]. For each food, the colour is based on its score: dark green (from −15 to −1 points); light green (from 0 to 2 points); yellow (from 3 to 10 points); light orange (from 11 to 18 points); and dark orange, almost red (from 19 points). These points are given to foods according to their content in energy (KJ/100 g or 100 mL), total fat (g/100 g or 100 mL), SFAs (g/100 g or 100 mL), sugars (g/100 g or 100 mL), protein (g/100 g or 100 mL), salt (g/100 g or 100 mL), fibre (g/100 g or 100 mL), Na (mg/100 g or 100 mL), and finally a group of fruits, vegetables, legume, nuts, and olive oil (%/100 g or 100 mL). The attribution of colours is achieved with a point scale from −5 (or lower) to 19 (or higher). Whole fruit is left out of this method, being a missing value [7]. The colour dark green, which receives an A value in Nutri-Score, being the healthiest value, was replaced by the score 5; the colour light green, which receives a B value, by the score 4; the colour yellow, which receives a C value, by the score 3; the colour light orange, which receives a D value, by the score 2; and finally, the colour dark orange almost red, which receives an E value (considered the least healthy), was replaced by the score 1.

#### 2.3.5. Multiple Traffic Lights from Ecuador

The system (from now on, “Ecuador”) used is in colours and horizontal bars: a red bar for products with “HIGH” fat (≥20 g/100 g), sugar (≥15 g/100 g), or salt (≥600 mg/100 g) content; a yellow colour bar for “MEDIUM” fat (>3 g - <20 g/100 g), sugar (>5 g - <15 g/100 g), or salt (>120 g - <600 g/100 g) content; and a green colour bar for “LOW” content in fat (≤3 g/100 g), sugar (≤5 g/100 g), or salt (≤120 mg/100 g) [38]. Subsequently, the colours were replaced by 1, 2.5, or 5 depending on whether they were red, yellow or green, respectively, and the arithmetic mean was taken.

#### 2.3.6. Chilean Warning Labels

The labels (from now on, “Chile”) are established when the nutrient limits established by the Food Sanitary Regulations are exceeded and/or the food has added sugars, sodium, or fat. The limits for the solid food stamp are marked as 10 g/100 g for the “HIGH SUGARS” stamp, 4 g/100 g for the “HIGH SATURATED FAT” stamp, 275kcal/100g for the “HIGH CALORIES” stamp, and 400 mg/100 g for “HIGH SODIUM” [2]. In this case, the presence of each seal was evaluated with a value of 1 (red) in each of the sections and with a value of 5 (green) for each absence of the seal. Subsequently, the arithmetic mean was calculated.

#### 2.3.7. Uruguayan Warning Labels

In 2018, Uruguay established that packaged foods must carry appropriate nutrition labelling (from now “Uruguay”), including warning stamps when some nutrient contents exceeded the established decree. Therefore, a warning stamp is shown when the sodium content is higher than 8 mg Na per 1 kcal or 500 mg per 100 g of food; the sugar content is higher than 20% of the total caloric value from sugars and 3 g per 100 g; the fat content is higher than 35% of the total caloric value; or the SFA content is higher than 12% of the total caloric value [39]. As with the Chilean warning labels, the presence of each seal was evaluated with a value of 1 (red) in each section and a 5 (green) for each absence of a seal. Subsequently, the arithmetic mean was calculated.

### 2.4. Statistical Analysis

**Figure 1:**
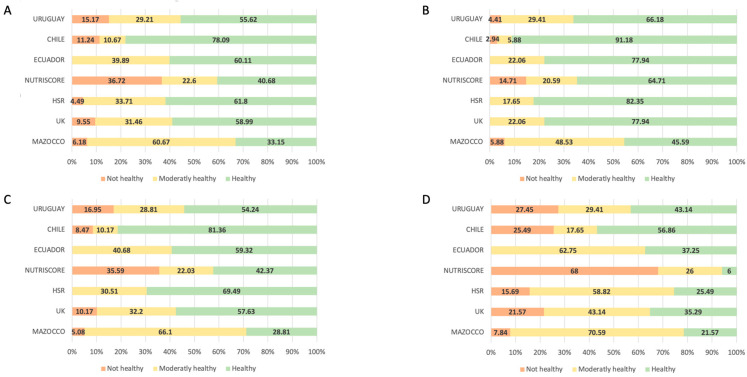
Graphical representation of the percentage results for dishes analysed according to the Front-of-Pack labels: Mazocco method, UK traffic light, HSR, Nutri-Score, Ecuador, Chile, and Uruguay. The colour RED indicates “unhealthy dishes”, YELLOW represents “moderately healthy dishes”, and GREEN signifies “healthy dishes”. (**A**) Overview of all dishes analysed; (**B**) analysis of first courses; (**C**) analysis of second courses; and (**D**) analysis of desserts.

In order to compare the different FOP labels, all the values needed to be numbered by a team of health personnel and statisticians (nutritionist, biochemist, and nurse). As the higher value was 5, this value was divided by 3 numeric sections to match the traffic light colours. In this ranking, the arithmetic means from 1 to 2.39 were considered to receive a value of “1” (the colour red in Figure 1), from 2.4 to 3.739 a value of “2.5” (the colour yellow in Figure 1), and from 3.74 to 5 a value of “5” (the colour green in Figure 1). The reproducibility and the proportion of observed agreements were calculated using the kappa index (K), which is given a value from 0 to 1. The value 0 means no agreement, 0–0.20 insignificant agreement, 0.21–0.40 medium agreement, 0.41–0.60 moderate, 0.61–0.80 substantial, and 0.81–1 almost perfect. A value of less than 0 indicates disagreement [42].

## 3. Results

Table 2 presents the nutritional composition of the dishes analysed in this study. Of the 533 dishes evaluated, 355 were repetitions, resulting in 178 unique dishes. On average, each serving contained 558.6 kcal. Among the three courses, the second course dishes had the highest average calorie content (795.9 kcal/serving), as well as the most fat (55.7 g/serving), saturated fat (10.2 g/serving), salt (1.4 g/serving), and sodium (925.6 mg/serving). On the other hand, the first courses contained the most fibre (11.0 g/serving), while desserts had the highest simple sugar content (33.7 g/serving).

Figure 1 shows the distribution of the analysed dishes that are “not healthy”, “moderately healthy”, and “healthy” in percentages. Panel A corresponds to the total number of analysed dishes. The Nutri-Score method identifies most dishes as “not healthy” (37%), followed by Uruguay (15%), while Ecuador does not identify any dishes in this category. Regarding the dishes classified as “moderately healthy”, the Mazocco method identifies 61% compared to only 11% using the Chilean FOP labelling. Within the “healthy” category, Chile has the FOP labels with the highest number of “healthy” dish ratings, with 78% of the dishes, in contrast to Mazocco method, with only 33% of the total dishes.

Panel B shows the FOP labelling method values for the first courses in the categorisation by dish. In this case, the UK, the HSR, and Ecuador do not identify any dish as “not healthy”, compared to 15% of those identified by Nutri-Score. Regarding the first courses within the “moderately healthy” category, the Mazocco method categorises 48% of first courses as moderately healthy, but Chile only classifies 6% in this category. Similarly, there is a contrast in the dishes classified as “healthy”, with 91% by Chile and only 46% by Mazocco method in this category.

Panel C shows the percentage of second courses categorised by the methods studied. Within the category “not healthy”, Nutri-Score finds 36% of dishes in this category, and both the HSR and Ecuador do not classify any dish. In the following category, “moderately healthy”, Mazocco method is the FOP method that classifies the most dishes (66%) in this range and Chile classifies the least (10%). Next, within the dishes considered by the FOP labels as “healthy”, Chile identifies 81% of dishes as “healthy”, with Mazocco method at the lower end, with only 29% classified in this way.

Panel D shows the percentages of the desserts categorised by each FOP method. Nutri-Score is the FOP method with the highest percentage of “not healthy” desserts (68%) and Mazocco method the lowest (8%). The Ecuador FOP method has no “not healthy” desserts. In the “moderately healthy” category, Mazzoco method is the FOP method with 71% and Chile with 18%. Finally, Chile is the FOP method that classifies most desserts as “healthy”, with 57% of the desserts in this category, and Nutri-Score classifies the least in this category, with only 6% of the desserts in this range.

Table 3 shows the K values to assess the proportion of agreement observed. Regarding the total number of dishes, the UK FOP labels show moderate agreement with Ecuador (K = 0.4921), Chile (K = 0.5234), and Uruguay (K = 0.404). Considering the first courses, the UK shows moderate agreement with Ecuador (K = 0.5723) and Uruguay (K = 0.4389). On the other hand, for second courses, there is no agreement between Ecuador’s FOP labels, Mazocco (K = −0.059), and Nutri-Score (K = −0.0802). However, Chile and the UK and Uruguay and Ecuador, with K values of 0.5155 and 0.426, respectively, indicate moderate agreement. Finally, in desserts, Ecuador shows a moderate agreement with the UK (K = 0.4096), which, in turn, also shows a moderate agreement with Chile (K = 0.5598).

## 4. Discussion

In this study, we compared the various Front-of-Pack (FOP) labels from different countries that are applied to evaluate the healthiness of cooked meals eaten away from home. We observed that the same dish can receive the healthiest or unhealthiest rating depending on the FOP labelling method applied. Nutri-Score emerged as the FOP label that classified most dishes as “not healthy”, whereas the label used in Ecuador does not classify any dishes in this manner. The labelling used in Chile identified more healthy dishes, and Mazocco method classified more dishes as yellow. The level of agreement between the FOP labels used was low, with moderate agreements found only between the UK and Chile and between Ecuador and Uruguay.

Comparing the different existing FOP labels is challenging due to varying methodologies and the number of variables considered. The classification shown by Mazzoco et al. [12] considers two factors, while Ecuador’s label examines three; however, they showed disagreement in the results of the second course analyses. The FOP labels that analyse four factors include the UK, Chile, and Uruguay, all of which examine SFAs, sugars, and Na (although the UK measures salt instead of Na). This similarity might explain their moderate agreement over the number of dishes analysed. Conversely, the HSR and Nutri-Score, which assess the most nutrients (eight and nine, respectively), exhibited a medium level of agreement with each other and the other FOP labels, despite both including consuming fruits, vegetables, and nuts.

Currently, the HSR is the most widely used labelling system in Oceania due to its comprehensiveness in measuring the quality of cooked dishes. However, our study suggests that there may be a more suitable FOP label for evaluating menus offered in a Mediterranean country like Spain, as it has yet to identify any unhealthy first and second courses and lacks concordance with the other FOP labels evaluated. The study by Dickie et al. (2020) assessed foods that Australians buy using the HSR and discovered that this system misleads consumers about the healthiness of many foods, inadvertently granting a “health halo” to almost three-quarters of ultra-processed foods displaying the HSR symbol [43]. Considering that the diet of Australian adults is characterised by the low consumption of fruits, vegetables, grains, energy, protein, fibre, and monounsaturated fatty acids [44], these findings stand in contrast to the positive effects attributed to the HSR FOP labelling system on Australian public health by other researchers [19,20].

Three Latin America FOP labels were analysed in this study: Chilean warning labels, Uruguayan warning stamps, and Ecuador’s multiple traffic lights. Despite originating from the same continent, an agreement was found only between Ecuador and Uruguay in evaluating second courses. Additionally, the Chilean warning stamps demonstrated a moderate level of agreement with the UK’s system for total dishes, as well as for main courses and desserts, despite differences in the presentation on food packaging (stamps vs. colour traffic lights). Both approaches have proven helpful in changing eating behaviours; however, warning stamps have been more effective in discouraging the purchase of unhealthy products [45,46] and reducing the consumption of sugar-sweetened beverages [5,46]. As these labels assess the content of kilocalories, sugars, sodium, and SFAs, their staged introduction has prompted a gradual reduction in the inclusion of these components in food products to avoid label inclusion [2,5,14]. Given the success and acceptance of the Chilean stamps, Mexico has adopted this system, adding a new stamp for “high in trans-fat” [46]. These findings are consistent with those of Senda et al. (2024), whose study emphasised the importance of continuous monitoring and the effective enforcement of the warning label regulations that have recently been implemented in Brazil. In addition, the study highlighted the importance of FOP labels in empowering consumers to make informed dietary choices consistent with their overall health and well-being [47]. Future studies could assess the potential implementation of these labels in other countries while adapting them to each country’s nutritional recommendations.

In Europe, we examined the UK’s FOP labelling system, its adaptation by Mazocco et al., and Nutri-Score. Although the UK labelling demonstrated greater agreement with the Latin American FOP labels, Nutri-Score presented discrepancies with the Ecuadorian labelling in evaluating second courses and desserts without any moderate agreement with other labelling systems. Despite the significance of its origin [11], numerous dietitians and nutritionists have expressed concerns about Nutri-Score’s classification of dishes. These concerns have led to the development of a perception and opinion study protocol involving food professionals to address the limitations, such as the positive classification of sugary foods, the negative classification of olive oil due to its fat content, and the interference of the food industry in this FOP method’s implementation [6]. These findings suggest that Nutri-Score may not be well adapted to the Mediterranean diet, in which extra virgin olive oil is a fundamental component [48]. Despite dietitians’ and nutritionists’ perceptions [6], Nutri-Score labelled a higher proportion of dishes as “unhealthy” (in red), particularly desserts. This was in contrast to the other FOP labels, such as Ecuador’s system, which did not rate any dishes as unhealthy, or the Mazocco method, which rated less than 8% of all dishes as unhealthy. Despite the perceived limitations, the prevalent use of Nutri-Score in evaluating foods in Mediterranean countries suggests a level of acceptance and the reliability of this system for assessing the healthiness of food in these contexts.

The labelling system proposed by Mazocco et al. [12] did not correlate with any other FOP label examined, likely due to its reliance on only two variables, making it less specific than the others. Since it does not assess sugar levels, it is the dish classification system with the lowest percentage of desserts labelled as “not healthy”, alongside Ecuador’s system (which does not classify any as not healthy). This limitation of its evaluation system may be one of the reasons why it has yet to be adopted as an applied FOP labelling method.

FOP labels generally enable users to differentiate between healthier and less healthy foods [8]. However, each FOP label varies in expression and design [10]. Various studies [10,11,12] have found that bordered labels with a contrasting and solid-coloured background, such as the UK FOP label, Nutri-Score, and Ecuador’s multiple traffic lights, improve visibility and legibility. Additionally, cautionary symbols (as seen in Chile and Uruguay) enhance the credibility of labelling and further assist consumers in identifying healthier foods [8,10] without differences among socio-demographic groups. Prior studies have investigated how food choices can change based on the labelling observed in supermarkets [8,10,11,14,19], with a maximum change of 2% [8]. However, supermarket products are used to prepare food, so this approach does not provide an accurate picture of final healthy meal consumption, as foods deemed “healthy” by an FOP label may be consumed in an “unhealthy” manner if the exceeded amount is consumed. To date, no study has compared the reproducibility and concordance of labels globally for cooked dishes, so the results of this study can help promote the need for new strategies to assess the healthiness of dishes in each country.

The results of this study highlight the complexity and variability in the nutritional classification of foods according to different Front-of-Pack (FOP) labelling systems. The low concordance between the FOP labels suggests that no universally suitable system exists for all regions or types of foods. This analysis demonstrates the importance of adapting the FOP labels to specific cultural and regional contexts, such as the Mediterranean diet in Spain. Furthermore, it underscores the need to develop more comprehensive and tailored strategies to assess the nutritional quality of prepared dishes, thereby promoting healthier food choices in each country. To enhance the effectiveness of FOP labels, policymakers should consider developing standardised yet adaptable FOP labelling systems that account for regional dietary patterns and nutritional needs. Moreover, implementing educational campaigns is necessary to improve the consumer understanding and use of FOP labels and regulatory support to ensure the consistent application and evaluation of FOP labels across different food contexts. Collaboration between international health organizations, governments, and food industry stakeholders could also help harmonise labelling practices and promote healthier food choices globally.

However, the practical application of FOP nutrition labelling, while potentially beneficial, presents several challenges for the industry, particularly in different out-of-home contexts. First, cooks who do not follow standardized recipes and are guided by taste or colour may not accurately evaluate the nutritional content of their dishes [25]. This would demand comprehensive training for staff to ensure the accurate creation and preparation of dishes, similar to the training required under the mandatory nutrition labelling law in the US [49]. Studies have shown that mandatory menu labelling can encourage the reformulation of dishes served by restaurants, thereby benefiting public health [26]. In settings such as buffets, where all-you-can-eat options are available, assessing the healthiness of consumed dishes is particularly challenging. Even if a dish is labelled as healthy, consuming it in large quantities would alter the nutritional intake, potentially misleading consumers [50]. There are also concerns among restaurant owners about the potential negative impact of providing nutritional information on their meals, as it could have a detrimental effect on their revenues [51], despite growing evidence that menu labelling does not significantly affect sales. Some restaurants have an extensive list of ingredients that increases the cost of their menus and complicates the calculation of FOP labels due to their variety. Cafés, for example, offer variations of the same product with different ingredients, such as different types of milk, sweeteners, or toppings, requiring multiple FOP labels based on consumer choices [25]. Moreover, the long-term implementation of menu and dish labelling involves additional costs and planning, especially when menus are frequently updated to ensure the efficient use of highly perishable foods. The unpredictable nature of the food industry often requires frequent menu changes, further complicating consistent FOP labelling [52].

The limitations of this study include the fact that the estimation of food item weights was based on data sourced from the School of Food and Catering of a university campus’s website. Despite efforts to account for weight changes during cooking, some challenges arose as several recipes needed to specify the amount of water added. This omission complicated the calculation of water evaporation during cooking, a necessary step for determining the actual weight per serving of the final cooked dish. To prevent this bias from affecting the accuracy of the FOP labels, the same amount of water evaporation was estimated proportionally for each dish. In addition, some of the food labelling systems examined in this study, such as the HSR, were initially designed for supermarket-packaged products, such as pre-cooked meals. While these systems are not intended to be used on some foods (i.e., unpackaged foods, foods not required to bear a nutrition information panel, etc.), they are also not excluded from being used on cooked dishes. On the other hand, FOP labels vary significantly in terms of design, colours, symbols, and content. Comparing labels with different formats can be complicated, and the results are not directly comparable, so the researchers in this study have tried to choose the most effective and most straightforward method, unifying the values of all FOP labels. However, each FOP label is designed to meet the nutritional recommendations of the study region. Applying different FOP labels to Mediterranean cuisine dishes can lead to erroneous nutritional values, again showing the need for qualitative methodologies that facilitate and unify the need for nutritional assessment.

## 5. Conclusions

This study highlights the variability and lack of consensus in the categorization of meals using different Front-of-Pack (FOP) labels worldwide, which can hinder consumers’ ability to make informed and healthier food choices. There is a clear need for an easy-to-understand FOP labelling system that resonates with a country’s socio-cultural context and culinary practices. Future research should focus on developing qualitative and context-specific labelling methods tailored to the specific culinary culture of each country. This approach would enable consumers to better evaluate the nutritional quality of out-of-home cooked dishes. By addressing the practical challenges of implementing FOP labelling in different dining contexts, public health outcomes could be significantly improved through the facilitation of healthier cooked food choices.

## Figures and Tables

**Table 1:** Overview of various Front-of-Pack (FOP) labels: labelling system and variables.

Front of Pack	Labelling System	Variables	Image Example
Mazzoco method [12]	Colour card	Na content and energy	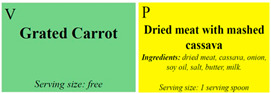
UK [37]	Colours: green, amber, and red	Fat, saturated fats, sugars, and salt	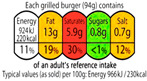
Health Star Rating [20]	Stars: from 0.5 to 5	Total energy, total sugars, and saturated fatty acids (risk components)Fibre, fruits and vegetables, nuts, legumes, and protein (healthy components)	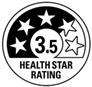
Nutri-Score [7]	Colours (dark green, light green, yellow, light orange, and red) and letters (A–E)	Energy, total fat, saturated fatty acids, sugars, protein, salt, fibre, Na, fruits, vegetables, legumes, nuts, and olive oil	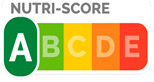
Multiple traffic lights from Ecuador [38]	Colours (green, yellow, and red)	Fat, sugar, and salt	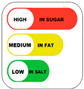
Chilean warning labels [2]	High content labels	Added sugars, Na, saturated fats, and calories	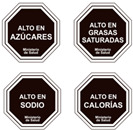
Uruguayan warning stamps [39]	High content labels	Na, sugar, fat, and saturated fatty acids	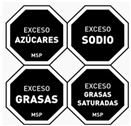

Na: sodium.

**Table 2:** Nutritional analysis of dishes examined by various Front-of-Pack labels.

	Dishes	Main Courses	Second Courses	Desserts
Total selected (*n*)	533	156	119	258
Duplicated (*n*)	355	88	60	207
Total analysed (*n*)	178	68	59	51
Total (%)		38	33	29
	Mean ± SD	Mean ± SD	Mean ± SD	Mean ± SD
Kcal/serving	558.6 ± 310	472.9 ± 167	795.9 ± 396	403.0 ± 148
Kcal/100 g	171.1 ± 101	134.7 ± 65.9	162.5 ± 93.6	231.5 ± 120
Fat (g/serving)	31.6 ± 30	19.7 ± 14.1	55.7 ± 40.9	20.3 ± 13.3
Fat (g/100 g)	9.8 ± 9.3	6.1 ± 6.0	11.6 ±10.0	12.6 ± 10.5
SFAs (g/serving)	7.8 ± 6.4	5.1 ± 4.1	10.2 ± 7.5	9.0 ± 6.5
SFAs (g/100 g)	2.8 ± 3.2	1.5 ± 1.5	2.1 ± 1.5	5.5 ± 4.6
Sugars (g/serving)	18.8 ± 15.7	14.8 ± 15.2	10.8 ± 7.1	33.7 ± 13.3
Sugars (g/100 g)	6.9 ± 7.3	3.5 ± 2.8	2.0 ± 1.3	17.2 ± 4.8
Fibre (g/serving)	7.3 ± 10.6	11.0 ± 8.2	5.9 ± 3.5	3.7 ± 16.0
Fibre (g/100 g)	2.0 ± 5.0	2.8 ± 1.9	1.1 ± 0.6	2.1 ± 9.1
Salt (g/serving)	0.7 ± 1.6	0.7 ± 2.0	1.4 ± 1.5	0.0 ± 0.0
Salt (g/100 g)	0.2 ± 0.3	0.2 ± 0.4	0.3 ± 0.3	0.0 ± 0.0
Na (mg/serving)	610.6 ± 697	662.3 ± 883	925.6 ± 539	171.8 ± 113
Na (mg/100 g)	167.8 ± 162	194.6 ± 216	186.7 ± 115	107.7 ± 95.2

Data are presented as mean and standard deviation (M ± SD). SFAs: saturated fatty acids.

**Table 3:** Association and observed proportion of agreement calculated using the kappa index (K) for various global Front-of-Pack labels.

Total Dishes (*n* = 178)							
	MAZOCCO	UK	HSR	NUTRI-SCORE	ECUADOR	CHILE	URUGUAY
MAZOCCO	—	—	—	—	—	—	—
UK	0.2049	—	—	—	—	—	—
HSR	0.2928	0.3367	—	—	—	—	—
NUTRI-SCORE	0.2318	0.1796	0.2252	—	—	—	—
ECUADOR	0.1554	**0.4921**	0.2723	0.0835	—	—	—
CHILE	0.1608	**0.5234**	0.316	0.176	0.3783	—	—
URUGUAY	0.1694	**0.404**	0.1945	0.1946	0.3759	0.3052	—
First Dishes (*n* = 68)							
	MAZOCCO	UK	HSR	NUTRI-SCORE	ECUADOR	CHILE	URUGUAY
MAZOCCO	—	—	—	—	—	—	—
UK	0.1521	—	—	—	—	—	—
HSR	0.2087	0.2168	—	—	—	—	—
NUTRI-SCORE	0.285	0.1508	0.2149	—	—	—	—
ECUADOR	0.07	**0.5723**	0.1247	0.1835	—	—	—
CHILE	0.0976	0.3083	0.1993	0.178	0.3083	—	—
URUGUAY	0.2554	**0.4389**	0.3798	0.1551	0.3337	0.1831	—
Second Dishes (*n* = 59)							
	MAZOCCO	UK	HSR	NUTRI-SCORE	ECUADOR	CHILE	URUGUAY
MAZOCCO	—	—	—	—	—	—	—
UK	0.0093	—	—	—	—	—	—
HSR	0.1782	0.2223	—	—	—	—	—
NUTRI-SCORE	0.2643	0.0332	0.0707	—	—	—	—
ECUADOR	* −0.059	0.3891	0.1227	* −0.0802	—	—	—
CHILE	0.072	**0.5155**	0.2441	0.0157	0.3591	—	—
URUGUAY	0.1324	0.3542	0.0499	0.1347	**0.426**	0.2761	—
Desserts (*n* = 51)							
	MAZOCCO	UK	HSR	NUTRI-SCORE	ECUADOR	CHILE	URUGUAY
MAZOCCO	—	—	—	—	—	—	—
UK	0.3816	—	—	—	—	—	—
HSR	0.3938	0.307	—	—	—	—	—
NUTRI-SCORE	0.0354	0.1056	0.1121	—	—	—	—
ECUADOR	0.3831	**0.4096**	0.2317	* −0.0099	—	—	—
CHILE	0.2508	**0.5598**	0.2557	0.1115	0.3343	—	—
URUGUAY	0.0743	0.3481	0.069	0.1223	0.3112	0.3493	—

*, value lower than 0. The value 0 means no agreement; values 0–0.20, insignificant agreement; 0.21–0.40, medium agreement; 0.41–0.60, moderate; 0.61–0.80, substantial; and 0.81–1, almost perfect. A value less than 0 indicates disagreement.

## Data Availability

The data presented in this study are available on request from the corresponding author. The data are not publicly available due to privacy.
